# Preferences of seniors living in selected Baltic Sea region countries towards the use of indoor public space furniture

**DOI:** 10.1371/journal.pone.0258676

**Published:** 2021-12-09

**Authors:** Beata Fabisiak, Anna Jankowska, Robert Kłos, Joan Knudsen, Catharina Gillsjö, Igor Kuprienko, Lyudmila Vidiasova, Anja Poberznik, Vineta Kreigere

**Affiliations:** 1 Department of Furniture Design, Faculty of Forestry and Wood Technology, Poznan University of Life Sciences, Poznan, Poland; 2 Department of Economics and Economic Policy in Agribusiness, Faculty of Economics, Poznan University of Life Sciences, Poznan, Poland; 3 Development Centre UMT, secretariat for Lifestyle & Design Cluster, Herning, Denmark; 4 University of Skövde, School of Health Sciences, Skövde, Sweden; 5 University of Rhode Island, College of Nursing, Kingston, Rhode Island, United States of America; 6 Project Development Division, ITMO University, Saint Petersburg, Russia; 7 e-Governance center, Institute of Design and Urban Studies,, Saint Petersburg, Russia; 8 Satakunta University of Applied Sciences, Faculty of Technology, Pori, Finland; 9 Art Academy of Latvia, Rīga, Latvia; Rīgas Stradiņa universitāte, LATVIA

## Abstract

Demographic changes can be observed all over the world. The number of seniors located in the societies of well-developed countries continues to rise. Both enterprises and governments need to be prepared for such changes. Consequently, public spaces need to evolve to reduce problems related to ageism and be friendly to all. Much attention is currently being paid to finding solutions for redesigning public spaces and adjusting them to the needs and requirements of senior citizens. To identify the preferences of seniors in relation to the characteristics of furniture in indoor public spaces, a survey study with 1539 respondents aged 60+ was conducted in Denmark, Finland, Latvia, Poland, Russia and Sweden. The gathered data were coded and implemented to the unified database. The statistical grouping method was used to recognize the characteristics of the needs and attitudes of seniors related to the use of public space furniture. The main variables taken into consideration in the analysis were the age and gender of respondents and their country of living. Among the most important findings are those indicating the necessity to provide the increased number of furniture for sitting in the public spaces and making sure they are not located too far away from each other. As the main disadvantages of public space furniture respondents indicated the lack of armrests or other solutions to facilitate getting up and/or sitting down, as well as profiled backrests that constitute solid support for the spine. The implementation of these data in the process of rethinking and redesigning public spaces may support the adaptation of indoor public furniture according to the requirements of a very large group of customers, namely, seniors.

## Introduction

Due to demographic changes, the structure of modern societies in highly developed countries is characterized by a growing number of senior citizens. Both public and private institutions need to be prepared for this social and economic challenge in order to provide best possible opportunities for health, participation and security to enhance quality of life as people age. This entails a great business opportunity exists for furniture manufacturing companies as there is a gap observed in the market. It is due to the fact that the senior population has never before been so numerous. Plouffe and Kalache [1] point out that as cities grow, their share of older residents also increases. Creating an environment that meets the expectations, desires and needs of seniors has become a major concern for social and public policy [2]. The processes of population aging and increased urbanization have encouraged researchers to recognize ways to develop a community that is accessible for all of its inhabitants [3]. To achieve this, the cooperation and efforts of urban planners, designers, architects, manufacturing companies and policy makers are needed [4]. Here the theory of the environmental gerontology comes into play [5] providing a better understanding of the interrelations between seniors and their physical-social environments [6]. This can be applied to private spaces like traditional housing (micro level of person-environment (p-e) interfaces), as well as public spaces e.g. neighborhoods, infrastructure, city districts (meso level) or urban, rural areas, regions or whole countries (macro level) [7]. Especially critical are the interventions on the level of public space e.g. enhancing safety and participation in a public life through means of barrier-free design [8]. The environment of the neighborhood shapes the quality of life [9, 10]. Neighborhood characteristics have also impact on behaviors and using of public space [11–13].

The World Health Organization (WHO) has developed an age-friendly city guide. The document highlights several domains that cities and communities can focus on to better adapt their structures and services to the needs of seniors. These domains include, among others, factors related to buildings [14]. The guide indicates that a designed for diversity physical environment can increase the independence of senior citizens and encourage them to overcome their obstacles [15]. The range of factors that determine a public space to be adapted to the needs of seniors is quite extensive, including adequate offers of housing, goods and services of daily use in neighborhoods; access to treatments; suitable places for both outdoor and indoor meetings; the ability to move independently; and, in particular, a solid social network [16]. One cannot forget that issues connected to the accessibility of local shopping and services, traffic and pedestrian infrastructure, neighborhood attractiveness, and public transportation also have a great impact on the level of activity and the quality of living of seniors [17]. Many researchers dedicated their works to recognition of the role of the local environment in promoting aging in place by creating livable communities and age-friendly cities [18–20]. Aging in place policy aims to have people remaining in their homes and communities for as long as possible. By seniors it is seen as an advantage in terms of a sense of attachment and feelings of security and familiarity in relation to both homes and communities [21]. Although much attention within the aging in place perspective is paid to the issues connected with preparation of home environment, one must not forget about the importance of neighborhoods and communities [22].

Public spaces are of crucial importance for sustaining the public realm. This is currently especially important, as modern societies no longer depend on town squares or piazzas for basic needs; therefore, designed for diversity public spaces are required for the social and psychological health of modern communities [23]. Consequently, great interest is currently being paid in regard to making public spaces, both outdoor and indoor, more accessible, safe and comfortable to as many citizens as possible. Most of the studies related to this aspect have concentrated on the recognition of seniors’ needs regarding outdoor public spaces, such as green areas [e.g., 24–26], or they have focused on street furniture and outdoor urban spaces in general [e.g., 27–31]. Some previous studies have focused on designing dementia‐friendly outdoor environments [32]. Other studies have been concerned with senior mobility issues in public spaces and reflecting on senior citizens’ points of view [33–36]. As far as indoor public spaces are concerned, most research has focused on building environments [37]. Facility management is the process of delivering and sustaining functions within building environments to meet strategic needs [38]. As mentioned above, it is essential to consider the needs of senior citizens during this process. Leung *et al*. [39, 40] highlighted that facility management in public housing should focus on the health and needs of seniors and provide a comfortable and convenient living environment for them. Nevertheless, studies of the impact of indoor public space design on the quality of living and attitudes towards indoor public spaces remain rare, especially in relation to the senior population.

The concept of indoor public spaces covers a wide range of public places that are often visited by senior citizens, including city halls, museums, libraries, restaurants, healthcare facilities, accommodation facilities such as hotels and culture or sport institutions, and many more. A significant part of the public space constitute waiting rooms. They are often considered as travel stops. Therefore, their important function is to provide the user with a place where he/she can comfortably gather energy for further activities. It is important to investigate design requirements of such public spaces especially in the context of their use by seniors, the vulnerable groups in late life who often experience increased tiredness [41]. This is among other due to the loss of muscle mass that at the age of 50–70 years is 8%, whereas, after the age of 70, this loss is averagely 10–15% [42]. Furthermore, what is even more significant is to enable users to relax without any feeling of discomfort or pain in waiting rooms, where people are often required to wait for a longer period of time, such as at train stations or airports. Often, unfortunately, waiting rooms are another example of undemocratically designed spaces, where seniors often encounter obstacles and lack of comfort. In view of the growth of the aging society, public institutions should pay more and more attention to the way in which this type of space is designed and equipped. However, the literature on the subject does not provide much guidance in this regard. More attention in this aspect has been dedicated to the patients preferences and design requirements for the waiting rooms in healthcare institutions [43, 44] e.g. in order to achieve stress reduction [45, 46], or while taking into consideration different cultural groups [47]. A number of studies can be found on increasing the waiting comfort in public spaces such as hospitals or administrative spaces [48], as well as on the influence of interior design of waiting areas on the perceived quality of service [49].

Based on the information collected during the literature review, it was noticed that there are only fragmentary recommendations that are useful in designing waiting rooms also for the senior population. Epprecht [50] recommends adding furniture of different construction to these spaces, i.e. a chair, a sofa for two people (love seat), which both serve as a seat for one person with a greater body weight, and as a regular sofa. Furniture in such spaces could create, for example, sets that would allow a family or a group of friends to gain more privacy and comfort; nevertheless, their diversity could also give more options to people suffering from health problems with subsequent difficulties in (living) daily life. Such a solution seems to result in much greater comfort than in the case of using chairs with a narrow seat located at small distances from each other. An additional clue that is highlighted by Epprecht [50] is that users do not want to sit back-to-back on chairs as they may accidentally touch their heads, which can be experienced as embarrassing and awkward.

Undoubtedly an important aspect of the indoor public space constitutes the furniture equipment. One must not forget that furniture pieces, especially e.g. the ones for sitting are among the pieces of furniture that are used directly–meaning the user’s body has a direct contact with the furniture. Therefore, they are of crucial importance to assure comfort, safety, and quality of living [51]. Thus, the aim of the current study was to recognize the needs and preferences of people aged 60+ concerning the use of indoor public space furniture to provide more insights for designers and furniture producers in their efforts to make indoor public spaces more senior-friendly.

## 2. Materials and methods

### 2.1. Study design and setting

The survey was performed within realization of international project BaltSe@nioR 2.0 aiming to provide new knowledge supporting creation of senior-friendly public spaces. The overall aim of the wide, international study performed within this project is also to indicate paths for potential areas of interest when solving problems that seniors face while functioning in public spaces. The results presented below refer to the part of the study concerning the preferences of seniors when using the public space furniture and their evaluation of disadvantages of furniture located in indoor public spaces.

To acquire the study data for the project realization the survey format was developed by experts representing various fields such as wood technology, design, geriatrics, and robotics from nine countries located in the Baltic Sea region. The survey format consisted of open- and closed-ended questions regarding the needs and problems seniors face while using public space furniture. The study was performed from April until September 2020. The closed-ended questions were followed by an open answer possibility which together with open-ended questions constituted a significant part of the study, enabling the respondents to describe in more detail their personal observations or provide comments about their doubts, worries or possible solutions that could be incorporated in the design and construction of furniture located in indoor public spaces.

### 2.2. Participants and survey procedures

A survey was conducted among people aged 60+. The study population comprised seniors living in Denmark, Finland, Latvia, Poland, Russia and Sweden. The research was conducted in the form of an electronic and paper survey distributed among seniors. The study was conducted in a written format with the help of a professional online survey platform (*4P*, *Warsaw*) in Poland, Survey Monkey platform in Denmark, Finland, and Anketolog service in Russia (https://anketolog.ru/). Paper versions of surveys were conducted in Sweden and Latvia with the support of university network and volunteer students. Project partners distributed the questionnaires using their own professional networks and personal contacts via e-mails, newsletters, websites, social media (Facebook, LinkedIn, etc.). Also various senior organizations have been contacted in order to facilitate reaching the target audience. They have redistributed the survey forms among their members. Participants were asked to fill in the questionnaires if they were 60+ and forward it further to whom it might concern. Such distribution of surveys did not allow for personal identification of individual respondents. The method used in the case of electronic surveys was a Computer-Assisted Web Interview (CAWI). All surveys were anonymous.

### 2.3. Analysis procedures

The questions analyzed in this paper were closed-ended questions both of single and multiple choice. The questions concerned the following:

- What type of public space do you prefer most?
- Is, in your opinion, furniture in indoor public spaces adapted to the needs of seniors?
- What type of furniture do you use most often in public space? (indoor)
- What activities would you like to be able to perform while using indoor seating furniture for example in the waiting room?
- Do you read newspapers / leaflets / books, etc. while waiting in the waiting room? If not, why?
- What weaknesses does furniture in the indoor public spaces have?

The gathered data were coded, implemented to the unified database and subjected to statistical analysis. The coding was done by transferring each item of the questionnaire into a variable reflecting the answer of the respondent. The answers provided under the open-answer option were analyzed separately. If various respondents indicated similar answers, the new codes were assigned to those responses, and that allowed for further comparative analysis of these data. Using the statistical grouping method, the characteristics of the needs and attitudes of seniors related to the analyzed subject were developed. The analysis was conducted using STATISTICA 13 PL software (Dell, Round Rock, TX, USA). Three main variables were taken into consideration–the age and gender of respondents, and their country of living.

The research question concerned the identification of the similarities regarding preferable senior-friendly features of indoor public space furniture in the 6 analyzed countries and age groups. The results may constitute inspirational source of knowledge for designers and furniture manufacturers to support them in creation of age-friendly products and through this facilitate senior daily functioning in indoor public spaces.

## 3. Results and discussion

Taking into account the percentage of completed surveys, a statistical analysis was conducted on the data obtained from 1539 seniors. Women constituted 49.6% of the sample population while men 50.4% (Table 1).

**Table 1:** Demographic profile of the respondents.

Overall sample (n = 1539)					
Age range [years]		Country		Gender	
60–64	5.7%	Denmark	10.5%	Female	49.6%
65–69	45.3%	Finland	7.3%	Male	50.4%
70–74	30.9%	Latvia	3.4%		
75–79	11.2%	Poland	65.0%		
80+	6.9%	Russia	7.7%		
		Sweden	6.1%		

Source: Authors’ own elaboration based on the performed survey research. Data available at www.baltsenior.com.

**Fig 1:**
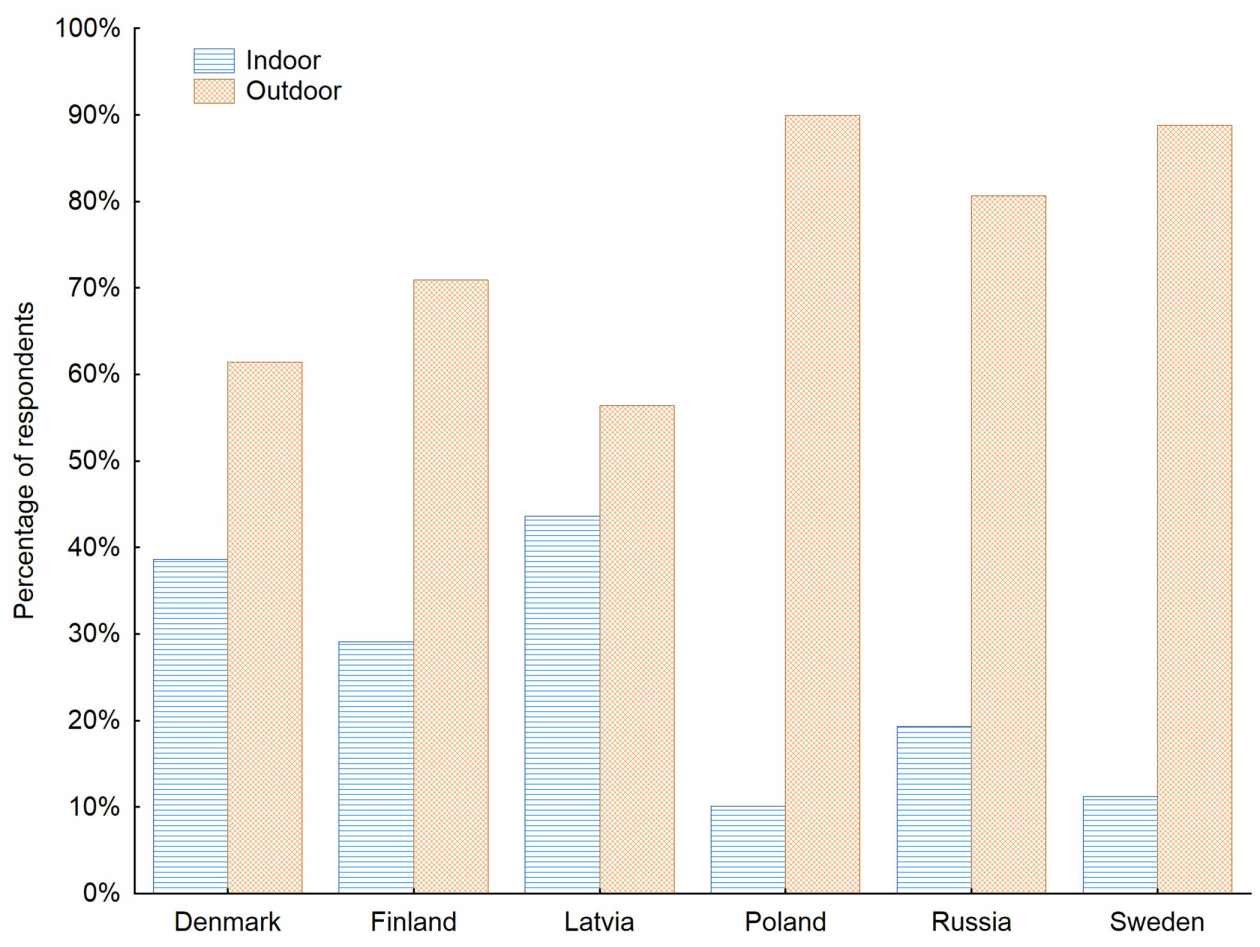
Opinion of respondents on the type of public space they prefer with regard to the country of living of respondents. Source: Authors’ own elaboration based on the performed survey research. Data available at www.baltsenior.com.

**Fig 2:**
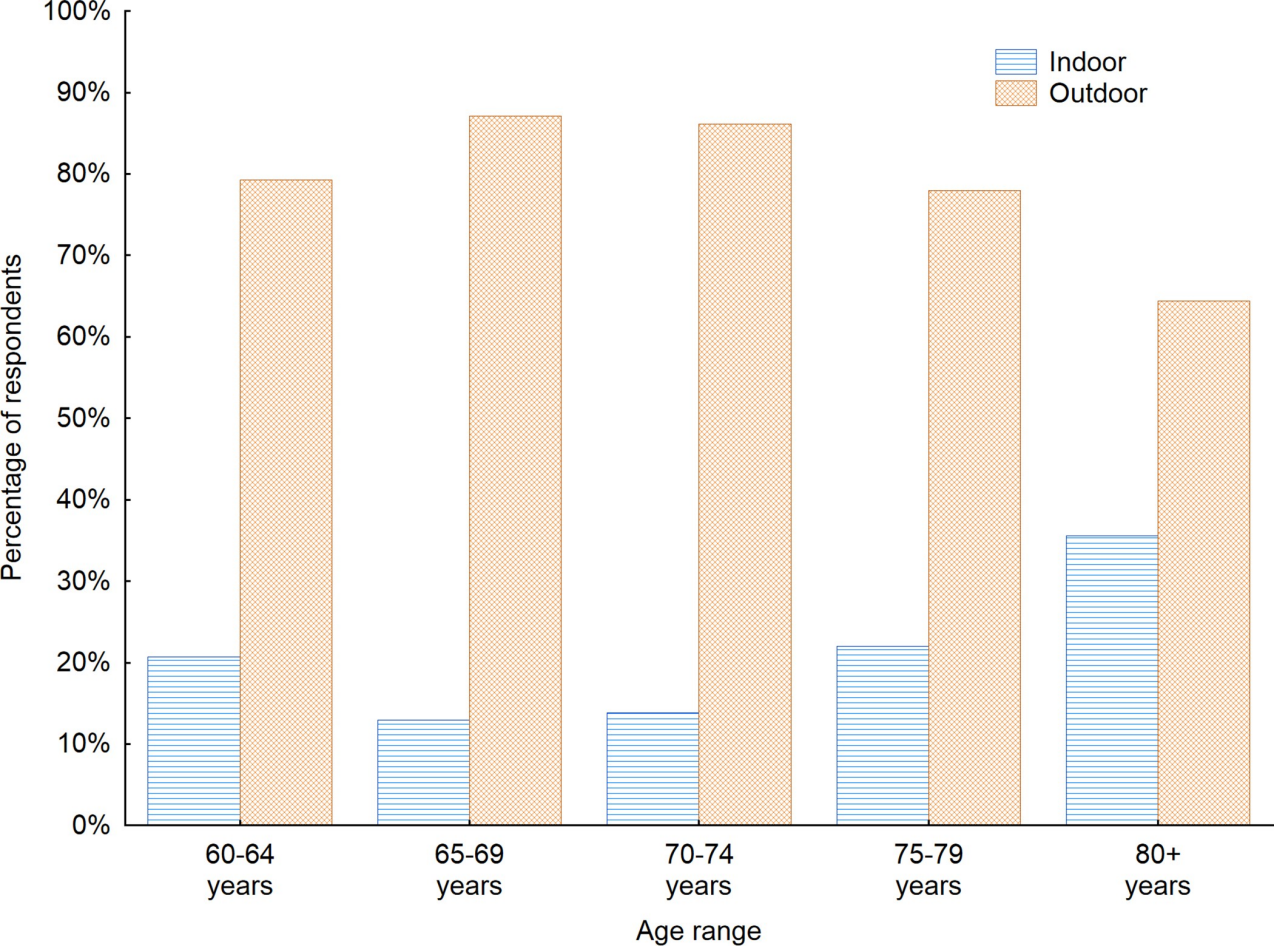
Opinion of respondents on the type of public space they prefer with regard to the age of respondents. Source: Authors’ own elaboration based on the performed survey research. Data available at www.baltsenior.com.

The first issue investigated was whether senior citizens prefer to spend their time in indoor public spaces, such as shopping malls, bus and train stations, museums, theaters, etc., or in outdoor public spaces, such as estates, parks, and promenades. In general, seniors preferred to spend the time in outdoor public spaces. In all analyzed countries, the majority of the responding seniors chose the outdoor public space as a more preferable one. The biggest number of seniors preferring indoor public space is seen in Latvia (43.6%), Denmark (38.6%) and Finland (29.1%) (Fig 1). As far as the age of the respondents is concerned the outdoor public space still remains the preferred one, however when we look at the older respondents the number of them choosing the outdoor public space decreases from 87.1% in the age range 65–69 years old to 64.4% in the age group of over 80 years old (Fig 2). When the gender factor was taken into consideration it turned out that slightly bigger percentage of men (86%) preferred the most to spend the time in outdoor public spaces. For women participants outdoor public space was preferred one for 80% still constituting the majority within the investigated sample.

The next issue investigated related to the preferences of seniors concerning the design and construction of public space furniture, was their opinion on the adaptation of such furniture to the needs and requirements of senior users. When the whole study sample was considered, it turned out that only 35.7% of the respondents reported that public space furniture is adapted to seniors’ needs. This result sheds light on how important the subject of the properly recognized needs of senior citizens is and how essential it is to incorporate those insights into the process of designing public spaces. This recognition is of crucial importance, as the wellbeing of senior citizens is also influenced by their participation in society and their ability to take an active part in public activities. Furthermore, public spaces, in addition to having functional value, foster a sense of identity with a city and have a wide influence on citizens’ activity patterns [52]. Wysocki [53] notes that a designed for diversity public space allows seniors to take full advantage of it, and this in turn has a positive effect on their quality of life. Moreover, he warns that a public space may become an environment full of obstacles and barriers for senior citizens if it is not adapted to their needs [54]. While considering the process of the adaptation of public spaces to seniors’ needs, it should be noted that people aged 60+ constitute the most heterogeneous group of users in terms of requirements. Thus, the universal design principles should be met to assure the best possible functionality [55–58].

**Fig 3:**
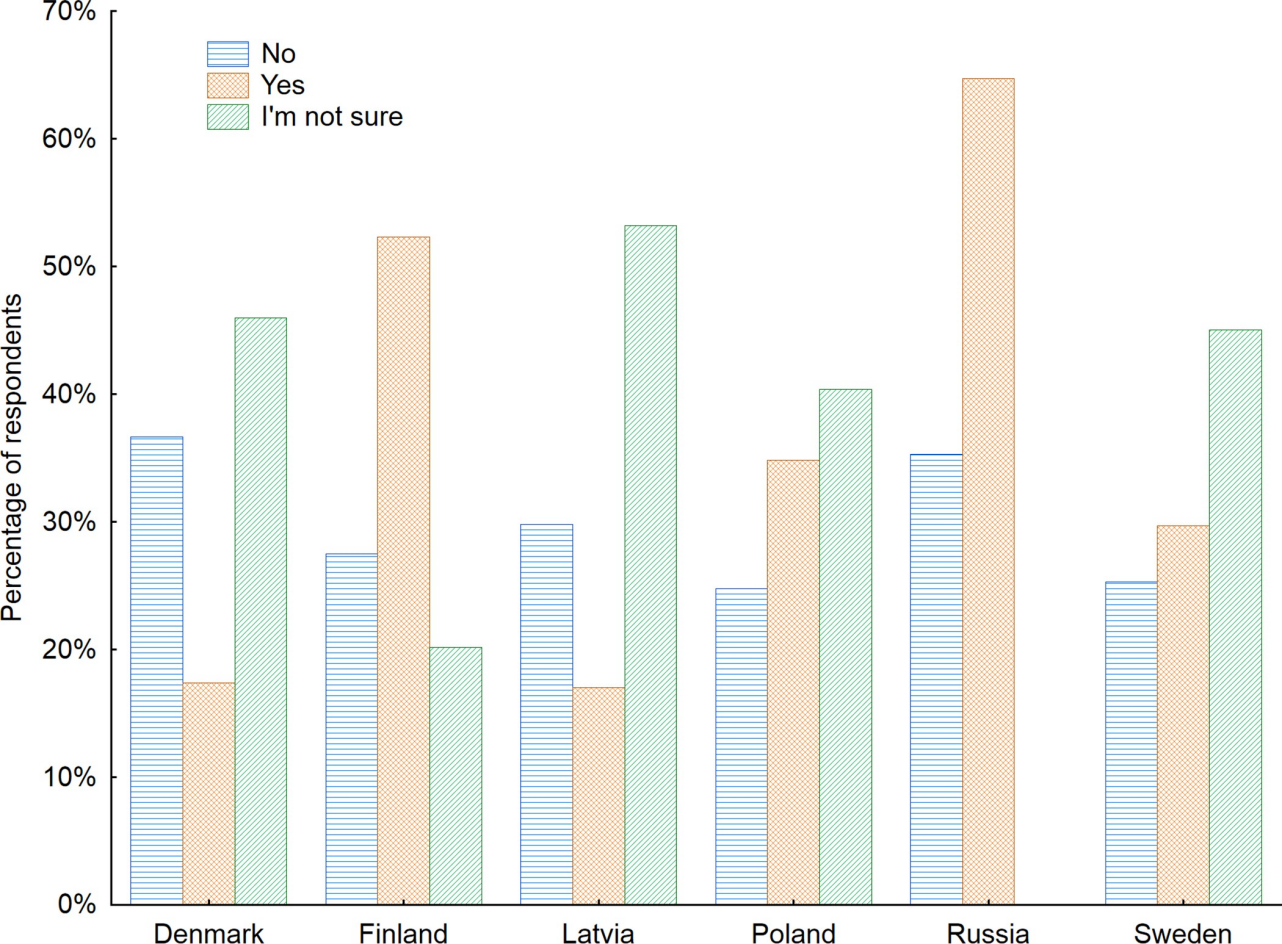
Opinion of respondents on senior-friendliness of indoor public space furniture with regard to the country of living of respondents. Source: Authors’ own elaboration based on the performed survey research. Data available at www.baltsenior.com.

**Fig 4:**
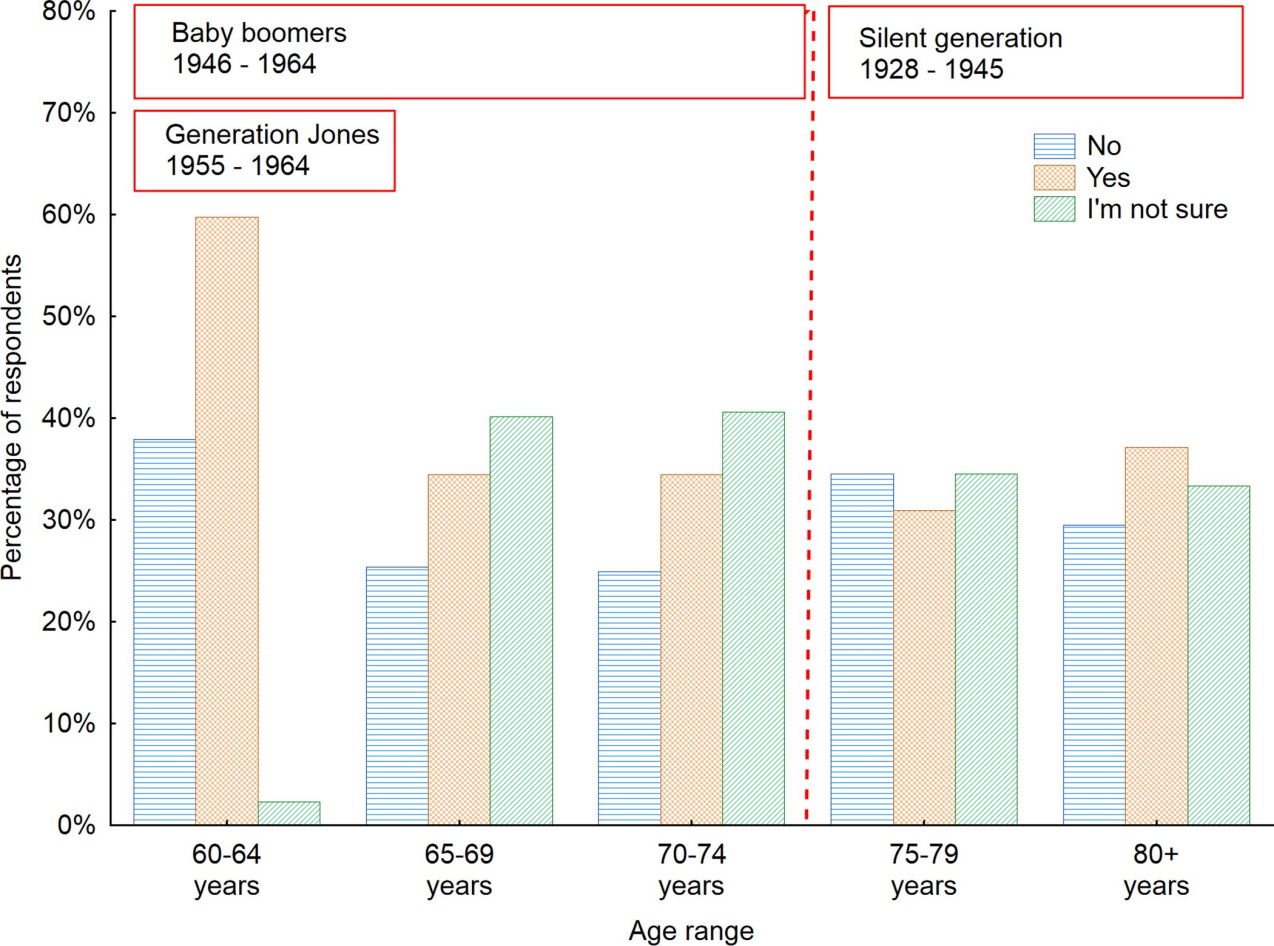
Opinion of respondents on senior-friendliness of indoor public space furniture with regard to the age of respondents. Source: Authors’ own elaboration based on the performed survey research. Data available at www.baltsenior.com.

A more comprehensive analysis was performed to recognize whether there are any similarities between the analyzed countries or aged group (Figs 3 and 4). The largest groups of seniors stating the public space furniture is adapted to their needs was observed in Russia (64.7%) and in Finland (52.3%). Nevertheless as far as Russian seniors are concerned it can be partly explained by the fact that survey participants have lived most of their lives in the Soviet Union and are not used to expressing strong grievances or complaints, especially about government jobs. A big surprise was a low percentage of Danish seniors considering the furniture in public spaces as senior-friendly– 17.4%. It is also worth to pay attention to the big number of respondents who were not sure whether the furniture is senior-friendly. This points out to the necessity of continuous actions aimed at raising awareness and presenting the newest possibilities in furniture and space design indicating how much influence they can have on the comfort and safety of citizens while using public space. Taking into consideration the gender factor revealed that men more often than women considered the public space furniture to be adapted to the needs of seniors. Nevertheless that was the opinion of only 39% of men respondents. This number for women was even lower and reached only 32%.

When investigating the age factor we can observe that various generations are represented in the sample population, as there are representatives of the Silent generation (born 1928–1945) and the Baby boomer generation. Furthermore, there is a need to acknowledge that Baby boomer generation is also divided into Early Boomer (born 1946–1954) and Generation Jones (born 1955–1964) [59].

**Fig 5:**
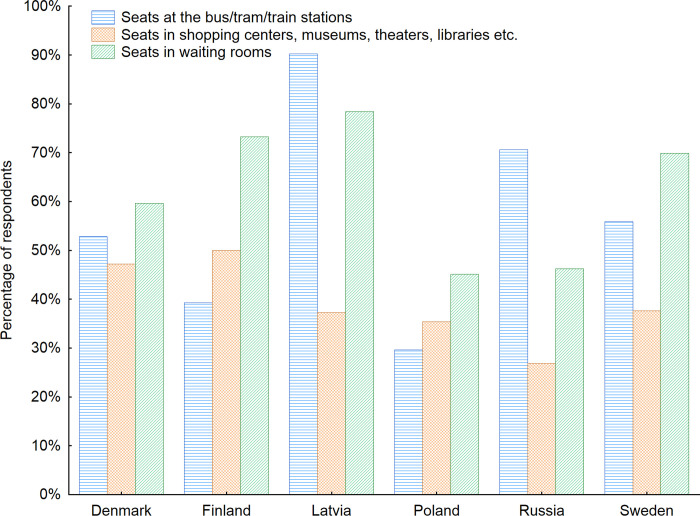
Types of indoor public space furniture used by the respondents most often with regard to the country of living of respondents. Source: Authors’ own elaboration based on the performed survey research. Data available at www.baltsenior.com.

**Fig 6:**
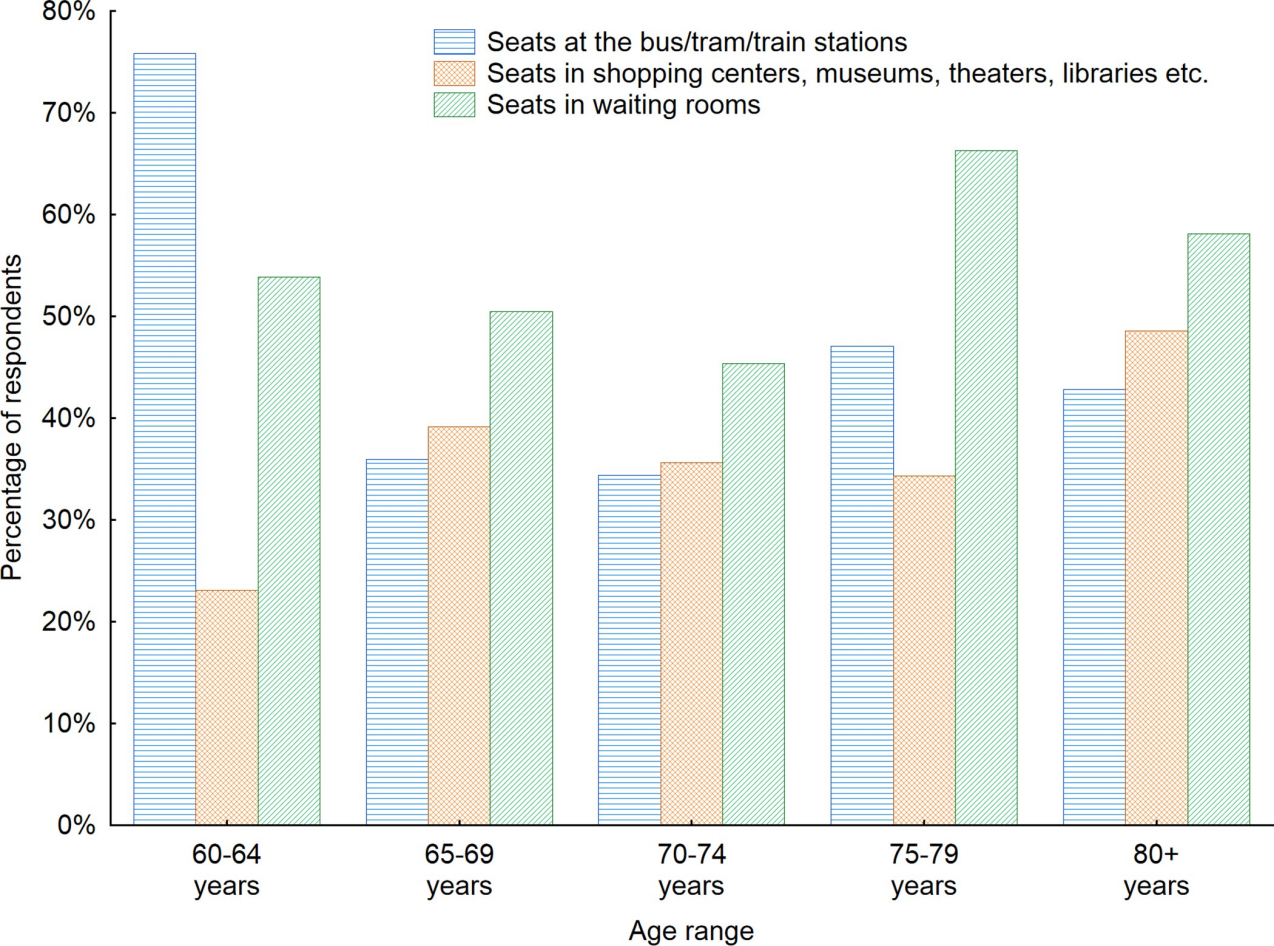
Types of indoor public space furniture used by the respondents most often with regard to the age of respondents. Source: Authors’ own elaboration based on the performed survey research. Data available at www.baltsenior.com.

In the next step, the analyze was focused on the types of furniture seniors use most often while being in the indoor public space (Figs 5 and 6). It turned out that the seats in the waiting rooms were used by the respondents most often. When the data divided into countries were analyzed, it showed respondents from Latvia and Russia used seats at the bus/train/tram stops most often– 90.2% and 70.6% respectively. In Sweden, Denmark, Finland and Poland seats in the waiting rooms were used most often. When the gender factor was considered it turned out that as many as 58% of women indicated the seats in the waiting rooms are among the ones used by them most often in the indoor public space. For men this number reached 45%. The seats at the bus/tram/train stations were listed as used most often by 46% of women and 33% of men.

**Fig 7:**
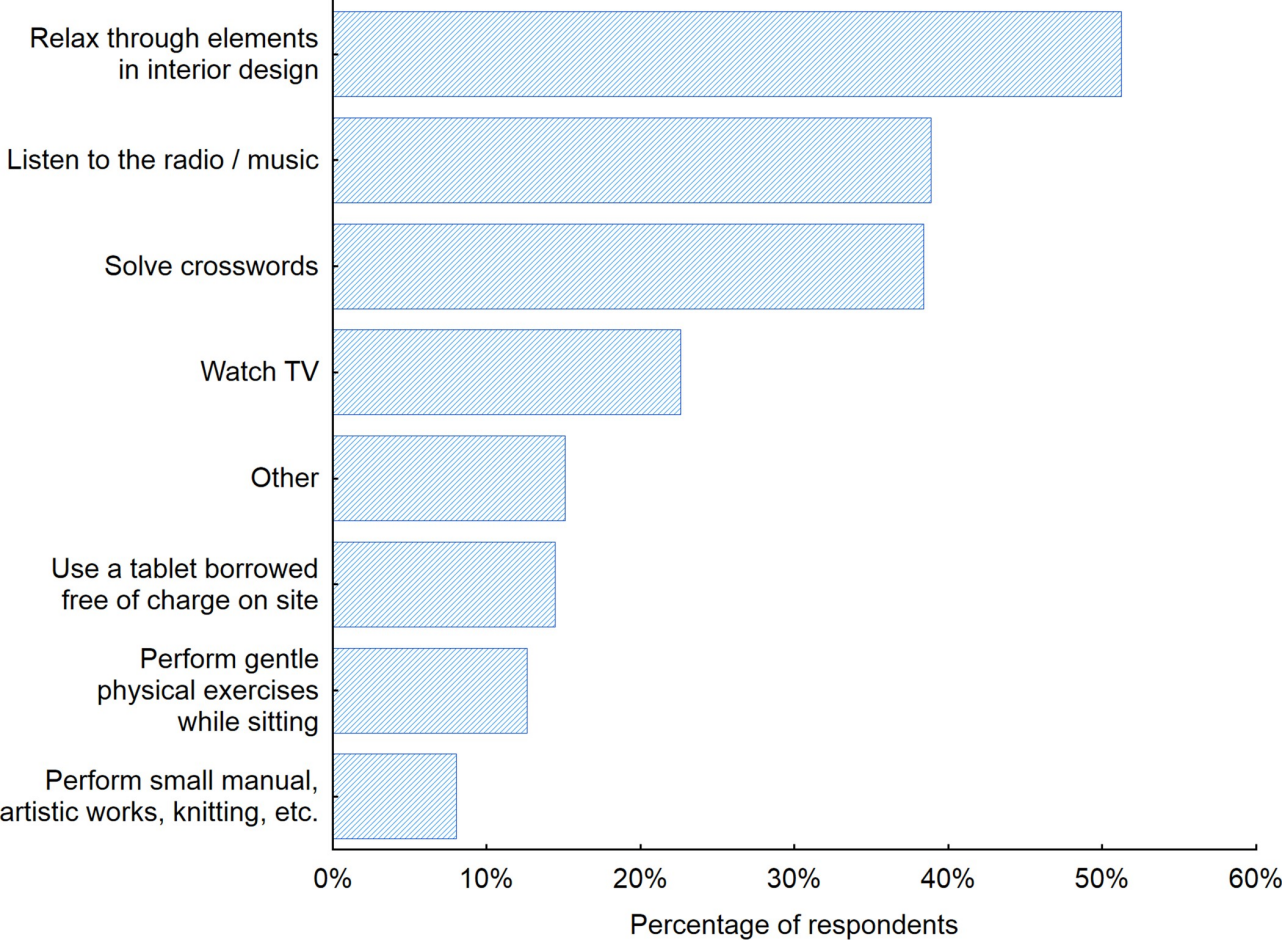
Activities that seniors would like to perform while using public space furniture in waiting rooms. Source: Authors’ own elaboration based on the performed survey research. Data available at www.baltsenior.com.

To recognize the preferences of seniors concerning indoor public spaces such as waiting rooms, we decided to investigate the obtained results in more detail. Thus, we asked the senior respondents what additional activities they would like to engage in while using indoor public space furniture for sitting, for example, in waiting rooms. We discovered that over half of the respondents (51.2%) would like to feel relaxed in waiting rooms thanks to the elements of interior design (Fig 7). This is a valuable hint for designers as it shows that the furniture and the interior elements should provide a sense of security and relaxation when employed in waiting rooms, for example. While analyzing the answers to the open-ended questions, we found that seniors would also like to enjoy conversations with other people in waiting rooms and would therefore like to use furniture and interior design elements that would facilitate interaction and communication between the users of a given indoor public space. Thus, the use of materials and solutions that improve the acoustic conditions within a given space would be very useful in regard to making it easier to hear other visitors.

**Fig 8:**
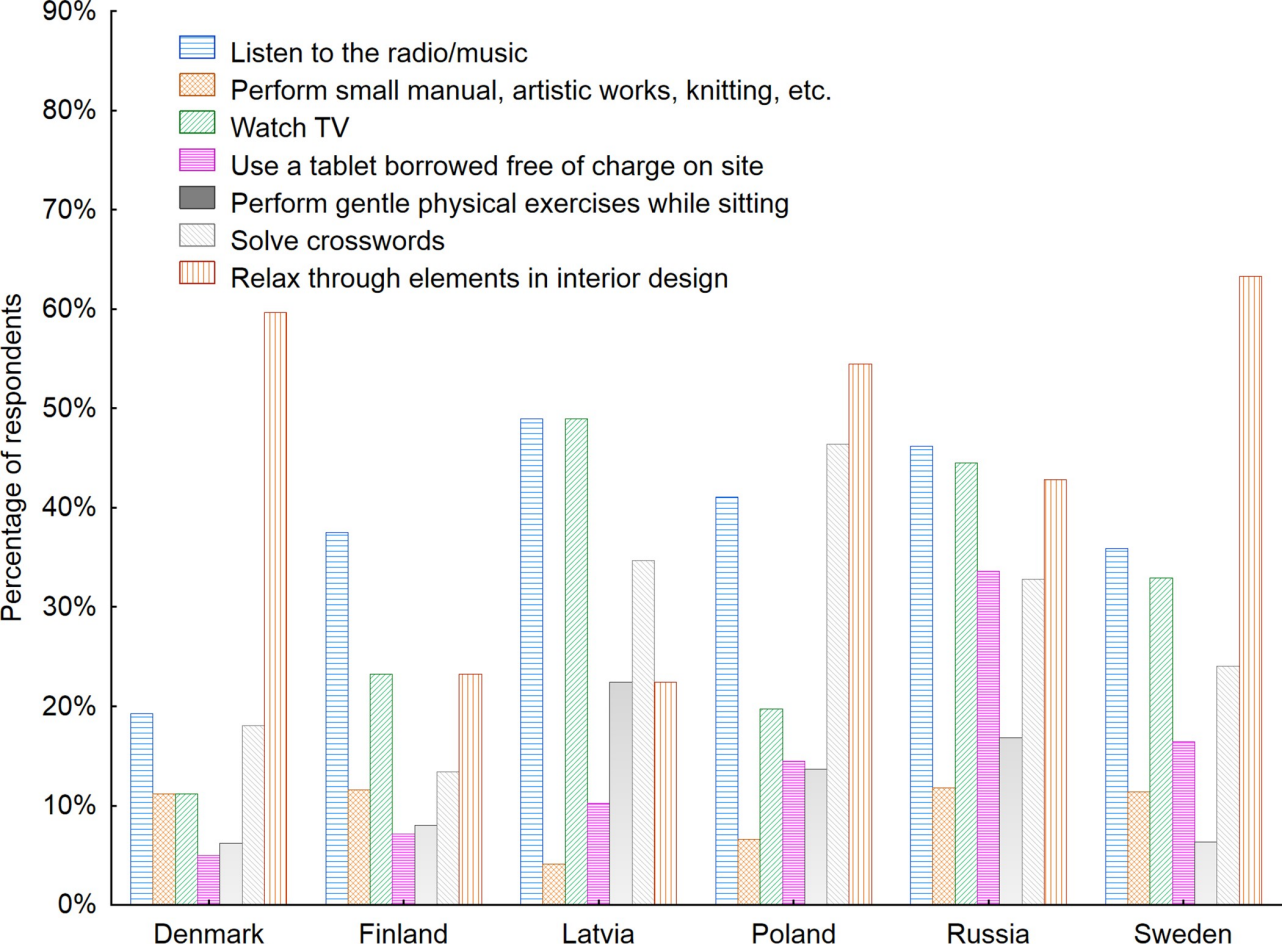
Activities that seniors would like to perform while using public space furniture in waiting rooms with regard to the country of living of respondents. Source: Authors’ own elaboration based on the performed survey research. Data available at www.baltsenior.com.

The possibility of relaxation during the waiting was the most important for the respondents in Sweden (63.3%), Denmark (59.6%) and Poland (54.5%) (Fig 8). Seniors in Russia and Latvia chose watching TV and listing to the music/radio as the most preferred ones. The analysis of the preferable activities seniors would like to perform while using public space furniture in waiting rooms revealed also that more men than women would like to listen to the radio/ music (46%) and watch TV (28%). While for women those numbers were 32% and 18% respectively. On contrary more women than men would like to perform small manual, artistic works, knitting, etc. (12% versus 4% for men). It is also interesting to note that the same number of men and women indicated that solving crosswords in the waiting room would be a preferable activity for them (38%).

**Fig 9:**
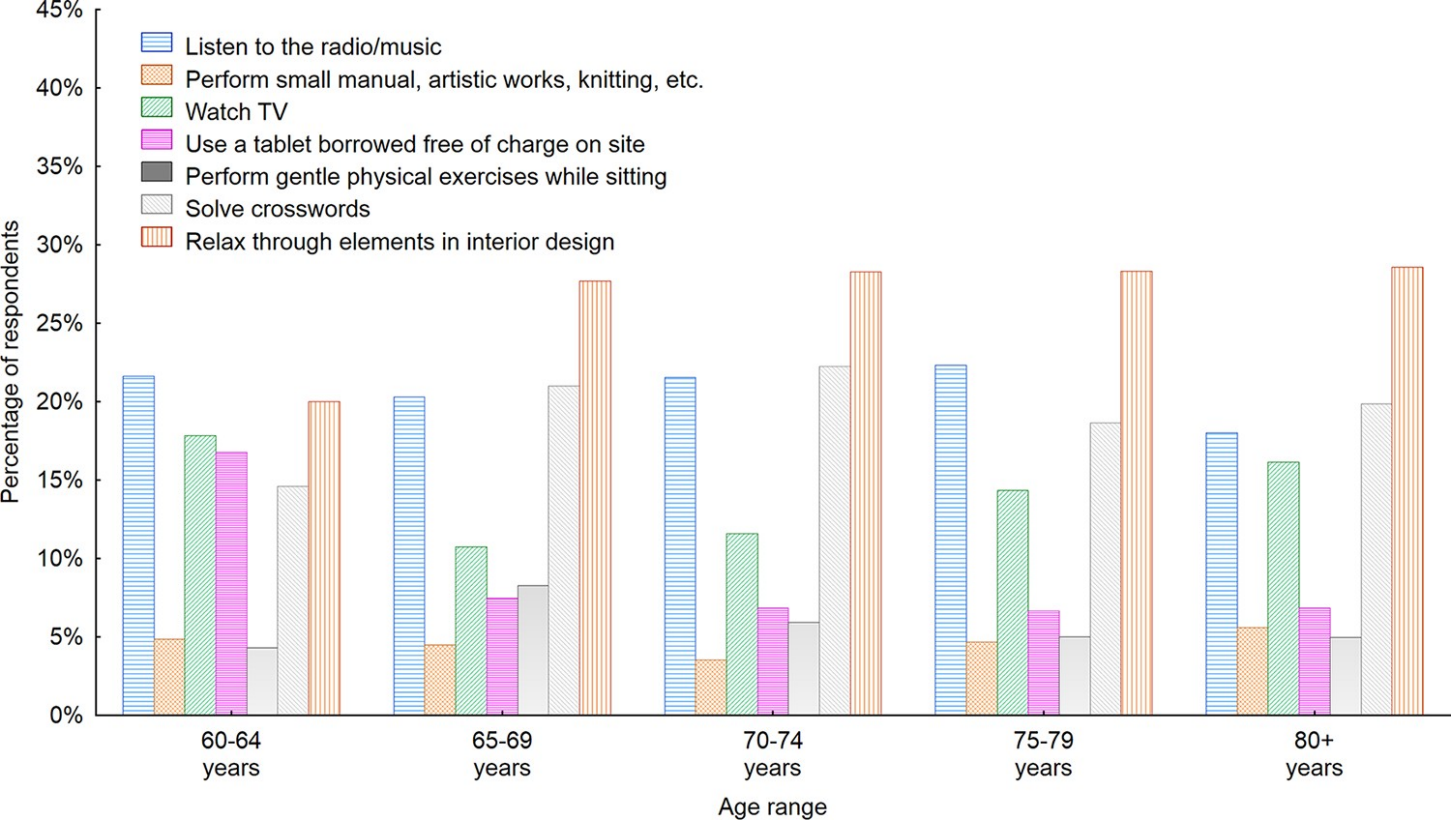
Activities that seniors would like to perform while using public space furniture in waiting rooms with regard to the age of respondents. Source: Authors’ own elaboration based on the performed the survey research. Data available at www.baltsenior.com.

Some interesting findings can be drawn from the data presented in Fig 9. First, an increasing interest in solving crosswords while waiting in indoor public spaces, watching TV and relaxing with the use of certain elements of interior design can be noted among increasingly older respondents. This finding gives designers and furniture producers an exciting starting point with regard to redesigning the way we think of waiting rooms and how to transform them into places where waiting is actually pleasant and enjoyable.

**Fig 10:**
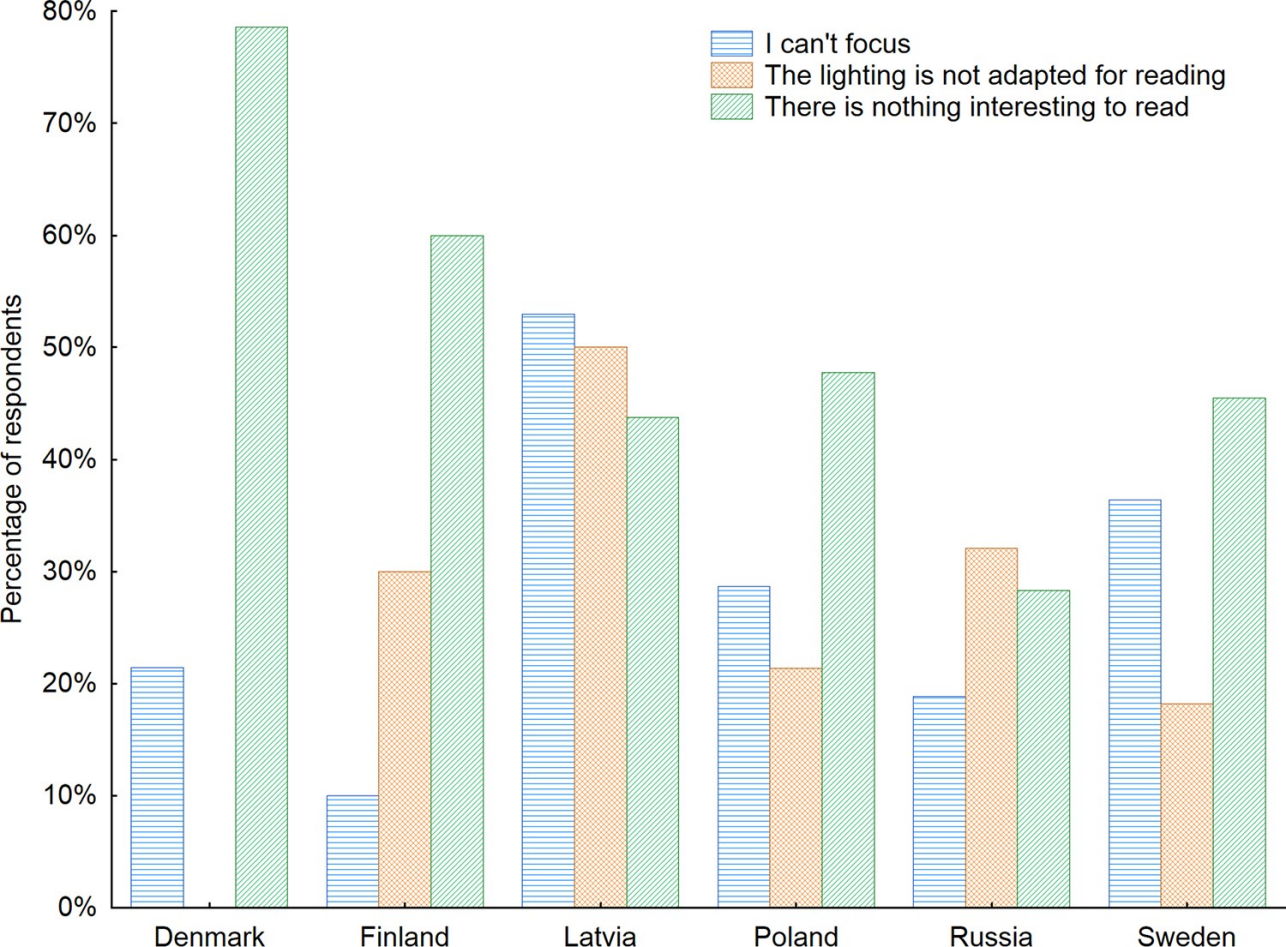
Reasons for not reading magazines and books while using furniture in waiting rooms with regard to the country of living of respondents. Source: Authors’ own elaboration based on the performed survey research. Data available at www.baltsenior.com.

**Fig 11:**
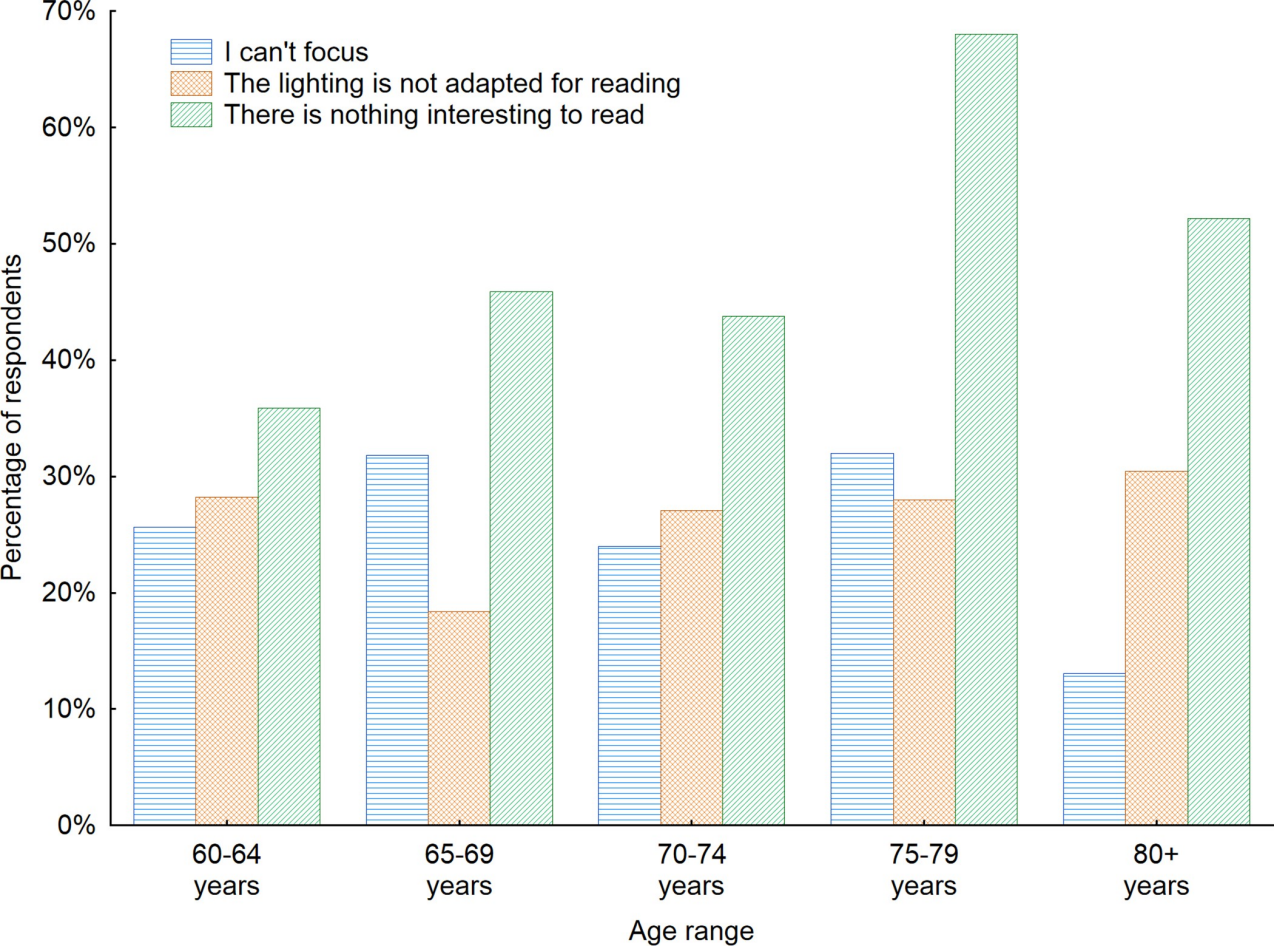
Reasons for not reading magazines and books while using furniture in waiting rooms with regard to the age of respondents. Source: Authors’ own elaboration based on the performed survey research. Data available at www.baltsenior.com.

Furthermore, among those who declared that they do not read newspapers, flyers, or books while using furniture in waiting rooms, we examined the reasons behind such decisions and wondered if those decisions were connected with the furniture and the interior elements of those indoor public spaces (Figs 10 and 11). Almost half of the respondents (46%) who reported not reading in waiting rooms admitted there were no interesting materials available to read. In open-ended questions, they pointed out that the materials are often very old and there is no use in reading old newspapers, advertisements or magazines. That confirms the results of the study of Arroll *et al*. [60] investigating the reasons behind the patient complaints about the oldness of most magazines in practice waiting rooms. In addition, 26.9% of the respondents declared that they were not able to focus on reading in such spaces, and 24.3% declared that the lighting in such spaces was not adjusted for reading. These are surely challenges that can be solved by designers and interior architects. Among other reasons, the majority of respondents admitted they do not bring with them additional glasses for reading. A good solution for meeting that need could be the provision of magnifying glasses installed in waiting rooms, similar to those that can be found in libraries and reading rooms. Furthermore, in open-ended questions seniors stated that in the times of COVID-19 pandemic they are afraid to touch the newspapers and books that were touched by others. They also highlighted that in many cases the publications are damaged and not clean which additionally evokes their fears of using them. Taking into consideration the gender factor indicated that women more often than men had problems with staying focused while reading in the waiting rooms (33%) and pointed out that the lighting often was not adapted for reading (30% of women). While for men this number was 23% and 20% respectively. On contrary men more often than women admitted they don’t read in the waiting rooms as there is nothing interesting to read (54% of men). The same opinion had 36% of women respondents.

**Fig 12:**
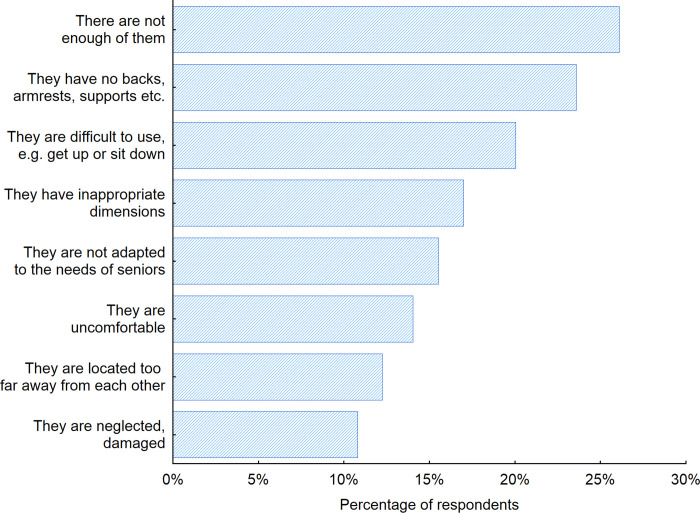
Disadvantages of indoor public space furniture according to the respondents. Source: Authors’ own elaboration based on the performed survey research. Data available at www.baltsenior.com.

To provide recommendations for the design of furniture used in indoor public spaces, a decision was taken to investigate in more detail the disadvantages of furniture located in such spaces. Most frequently, seniors reported that the amount of furniture provided within a given space is too small and thus they are not able to use furniture in the indoor public spaces every time they would like to (26.1% of the respondents) (Fig 12). The respondents also expressed concerns about certain construction features of the furniture; in many cases, the furniture either lacks armrests or support to help seniors get up easier (20%) or the backrests are not shaped in a way that supports the lumbar spine to increase the comfort of sitting (23.6%). The respondents also pointed out that the seats are often too flat and too hard to sit on.

**Fig 13:**
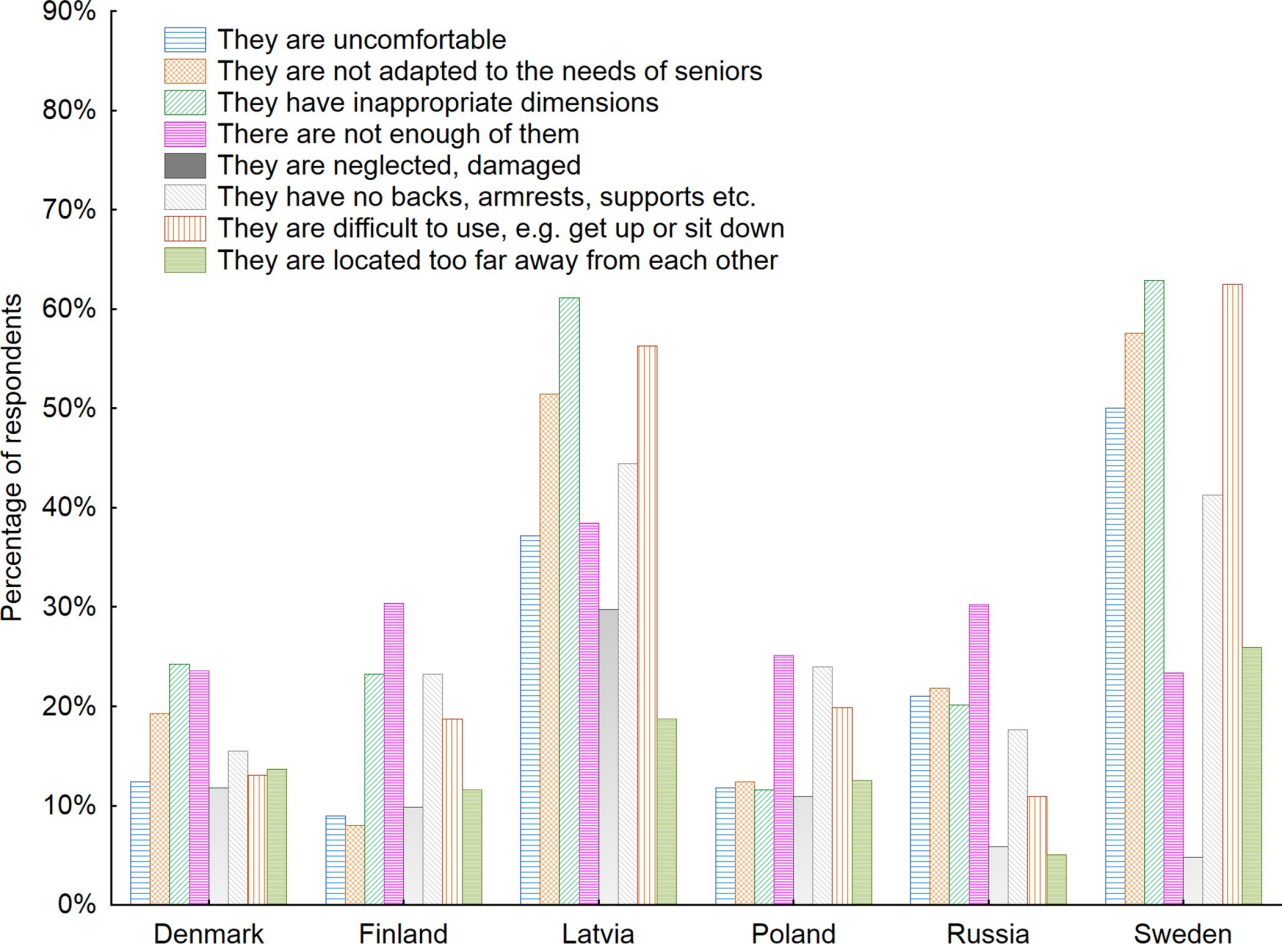
Disadvantages of indoor public space furniture according to the respondents with regard to the country of living of respondents. Source: Authors’ own elaboration based on the performed survey research. Data available at www.baltsenior.com.

Over 60% of respondents in Latvia and Sweden admitted that the furniture located in indoor public space have inappropriate dimensions–the seats are located too low or too high or they are too narrow and thus they are difficult to use for example get up or sit down (Fig 13).

**Fig 14:**
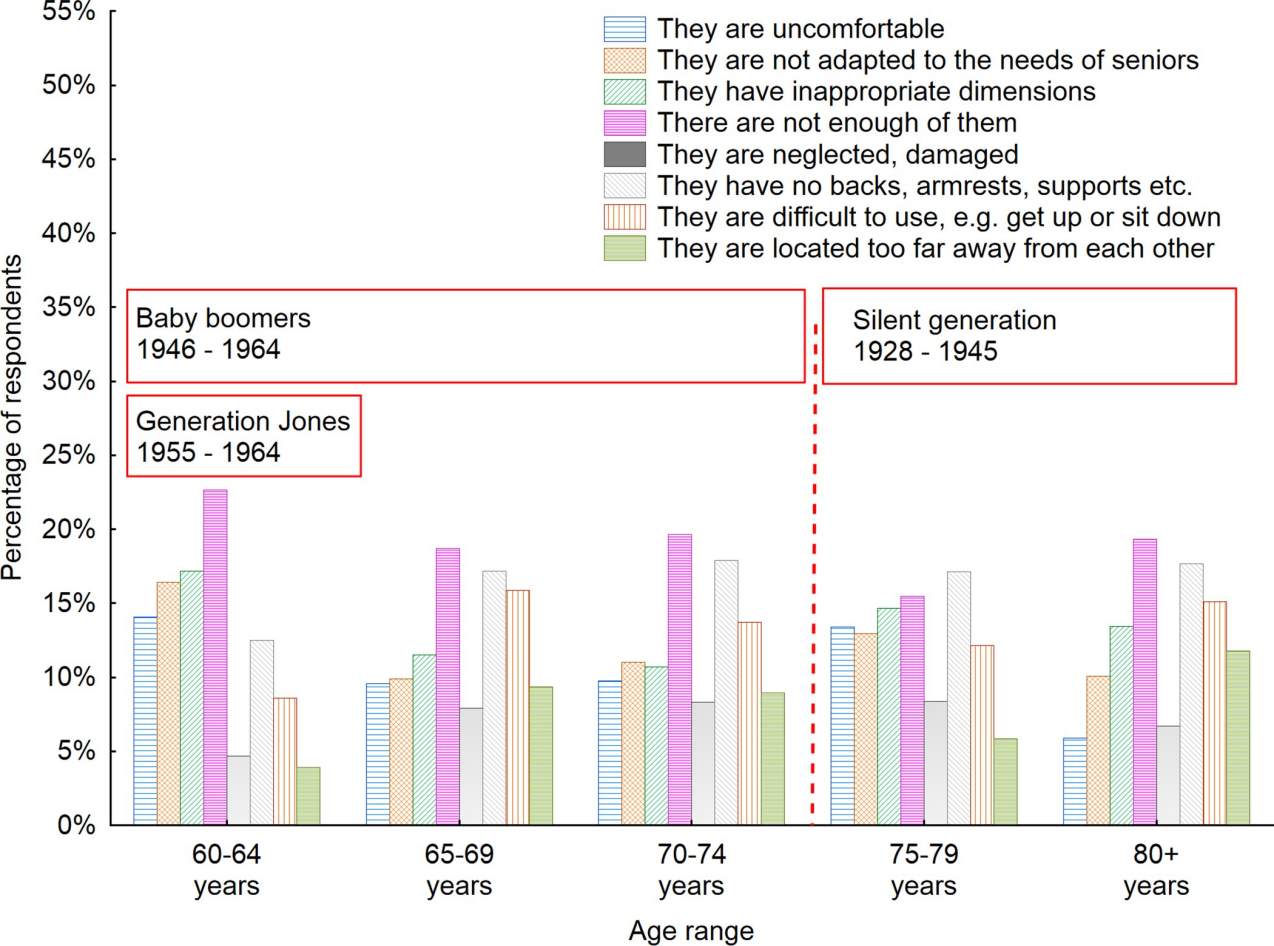
Disadvantages of indoor public space furniture according to the respondents with regard to the age of respondents. Source: Authors’ own elaboration based on the performed survey research. Data available at www.baltsenior.com.

As understanding the attitudes of seniors towards the furniture they come in contact with in indoor public spaces is crucial in regard to making further improvements to senior-friendly public spaces, it was decided to investigate how those opinions change according to age. Thus, a statistical analysis was conducted of this issue with regard to the age of the respondents using the statistical grouping method (Fig 14).

As mentioned earlier various generations were observed in the investigated sample. The generations differ between each other and this is also reflected in the results obtained. Even though the oldest groups of users were supposed to feel the greatest discomfort while using public space furniture, the representatives of the Silent generation did almost not complain at all. Among the 80+ age group, the largest percentage paid attention to the fact that there is not enough furniture, so they are not able to use it every time they need it. They also noticed that the furniture in indoor public spaces usually does not have backrests, armrests and other supports to increase the comfort of using the furniture. When the answers of Baby boomer generation are analyzed, it can be noticed that seniors in this generation complained the most about the number of pieces of furniture in indoor public spaces. Among respondents in the Baby boomer generation, the low number of people who considered the lack of backrests, armrests and other supports as one of the biggest disadvantages of indoor public space furniture increased with an increase of the age of respondents.

When the gender factor was considered, it turned out that more men than women among the weaknesses of pieces of furniture in the indoor public spaces list the insufficient number of them (28%) and the lack of backrests, armrests, supports etc. (26%). Whereas more women than men pointed out the furniture is not adapted to seniors’ needs (16%), it has inappropriate dimensions (19%) and it is difficult to use, e.g. get up or sit down (21%). It is worth to notice that all 3 above mentioned weaknesses of the furniture are among others connected with the anthropometrics of a human body that is significantly different for men and women especially in later stages of life [61–65].

As far as the limitations of the study are concerned, it needs to be stated that, due to the realization of the study also in the form of face-to-face interviews that might have been more suggestive with how the questions were asked than the electronic versions, some subjectivity could have influenced the participants’ answers. Furthermore health-related and other socio-demographic issues such as income, education, ethnicity or sexuality were not taken into consideration. Limitations also include the large variety in the number of respondents from the 6 countries as well as the possibility for various interpretations of questions when the survey questions are translated into many different languages. Nevertheless a big number of participants of this study allows for receiving a valuable insights into the analyzed subjects. Additional research must be conducted to further investigate the cultural aspects’ effects on the detailed preferences of seniors in different countries. Additional factors connected with the space such as type of the public space, it’s size, availability of staff, length of the stay in the space will be investigated in further studies. Thus, it can clearly be stated that the manuscript, although being a valuable inspiration source about the preferences of seniors should not discourage for including seniors into the design process. Inclusive design brings in enormous possibilities for finding the solutions that meet the needs of wider group of people and is always a beneficial approach [66, 67].

## 4. Conclusions

The obtained results provide valuable insights for designing more senior-friendly furniture for indoor public spaces. This is especially crucial in the context of the results of Avlud *et al*. [68] who indicated that tiredness in daily activities is also a consequence of age-related physiological and biological changes that are not entirely disease-based. This tiredness may cause seniors to need to sit and rest more frequently; thus, respondents pay attention when there is not enough furniture, that they are able to use in a public space or if such furniture is located too far away. A good solution in these cases might be making various types of folding chairs available in indoor public spaces. Such chairs do not take up much space but are available whenever needed. The second group of furniture features the respondents pay attention to is connected with functionality, which demonstrates adaptation of furniture to the physical and psychological needs of the users [69, 70]. Respondents pointed out e.g. inappropriate functional dimensions of furniture located in public space. Seniors indicated that public space furniture rarely has features that make the furniture senior-friendly. They most often pointed out the lack of armrests or other solutions to facilitate getting up and/or sitting down, as well as profiled backrests that constitute solid support for the spine. Another significant conclusion is also connected with the design of furniture and with the interior design elements of waiting rooms. In regard to this aspect, we observed among the senior respondents a growing interest in solving crosswords while waiting or maintaining interactions with other visitors, as well as relaxing in a supportive atmosphere within the indoor environment. Another important issue is connected to a huge opportunity that can be seen in designing furniture that both facilitates mutual interactions and improves the acoustic conditions of indoor spaces to enhance the customer experience for those who have hearing problems. Another possible design direction is connected with the implementation of wooden elements, plants and color schemes that enable easier relaxation in indoor public spaces.

Providing senior-friendly public spaces that promotes seniors’ wellbeing and quality in life constitutes a large design challenge; however, with a detailed analysis of users’ needs and a deep understanding of seniors’ attitudes and requirements, it is possible to redesign indoor public spaces in such a way that senior citizens are able to fully participate in their communities and maintain an active social life. Senior-friendly furniture located in various types of indoor public spaces can encourage senior citizens to use public spaces more frequently and seize the opportunity to function more independently, which is beneficial for the both the individual and society as a whole.
